# Rescue of Iatrogenic Spiral Dissections Using an Intravascular Ultrasound-Guided Parallel Wire Technique

**DOI:** 10.1016/j.jaccas.2024.103121

**Published:** 2025-02-05

**Authors:** Harish Sharma, Arif Khan, Benjamin Wrigley, Sohail Q. Khan

**Affiliations:** a Institute of Cardiovascular Sciences, University of Birmingham, Edgbaston, Birmingham, United Kingdom; b Department of Cardiology, University Hospitals Birmingham NHS Foundation Trust, Edgbaston, Birmingham, United Kingdom; c Department of Cardiology, Royal Wolverhampton Hospitals Trust, Wolverhampton, United Kingdom

**Keywords:** aorto-ostial dissection, coronary dissection, IVUS, spiral dissection

## Abstract

Percutaneous coronary intervention carries a risk of iatrogenic catheter dissection. A spiral aorto-ostial dissection can completely occlude the vessel and cause ischemia with significant hemodynamic compromise. The mortality from such dissections is approximately 6.5%. The situation can be rescued percutaneously by stenting the true lumen open, but this relies on having a wire within the true lumen. Large dissections often have a small true lumen that is hard to wire and a large false lumen that wires easily. There is a paucity of literature outlining the necessary steps to achieve procedural success. This case series includes 2 spiral dissections and demonstrates a step-by-step approach to manage this situation successfully.

## Visual Summary

**Figure:**
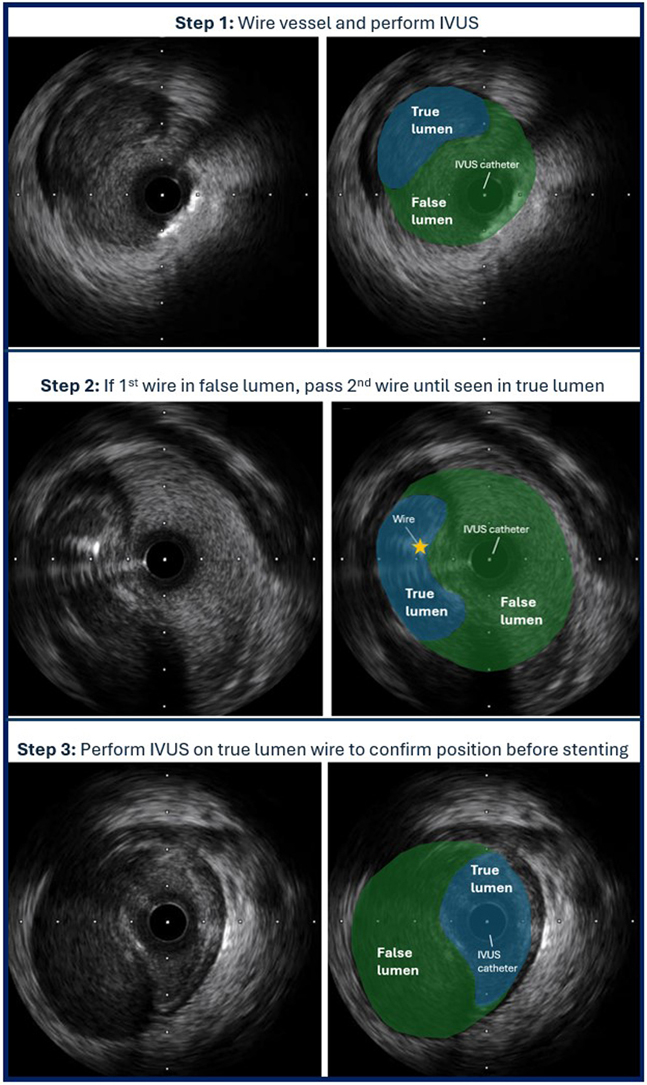

Iatrogenic catheter dissections are rare, with an incidence of 0.1%,^1^ but they carry a significant risk of mortality.^2^ Aorto-ostial dissection flaps often spiral throughout the vessel and result in ischemia from impaired flow or even vessel occlusion from compressive hematoma. This scenario can be percutaneously rescued by wiring the true lumen and stenting the vessel at the inflow of the dissection. Unfortunately, there is a paucity of literature outlining the critical steps necessary to carry out this procedure successfully. Here we describe 2 cases of catheter-induced spiral dissection, in the right and left coronary systems. The cases demonstrate the procedural stages and highlight the importance of intravascular ultrasound (IVUS) in achieving successful outcomes.

**Take-Home Messages**

• Catheter-induced spiral dissections are rare but carry a significant risk of mortality. On recognition of this disorder, avoid propagation by not performing any further contrast injections.
• The vessel should be promptly wired and IVUS used to check the position of the wire.
• If the wire is in a false lumen, then the catheter should be disengaged (particularly in aorto-ostial dissections), and a second wire should be passed into the vessel while maintaining live IVUS imaging from the false lumen wire. Once the second wire is identified in the true lumen, IVUS is performed on this wire to confirm its position, and then the vessel can be stented to restore and maintain antegrade flow.

## Case 1

### Background

A 51-year-old man was admitted with a 1-week history of chest pain. He had a background of hypertension and smoking. His electrocardiogram (ECG) did not show any ischemic changes, but serial high-sensitivity troponin levels were elevated at 263 and 1,140 ng/L. A computed tomography angiogram ruled out an aortic dissection.

### Procedure

**Figure 1:**
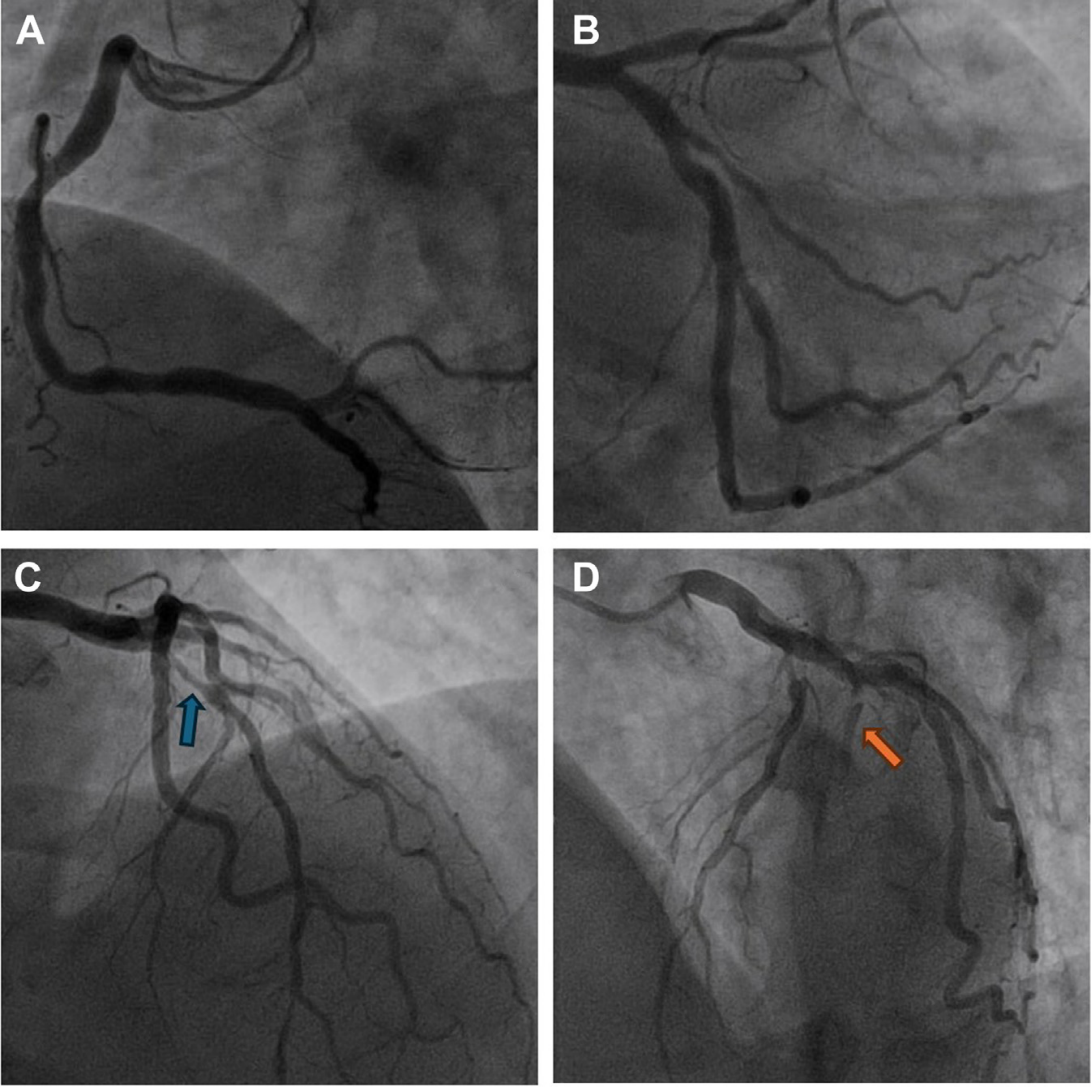
Initial Coronary Angiography (A) Right and (B to D) left coronary vessels. The left coronary vessels are shown in the (B) posteroanterior caudal view, (C) posteroanterior cranial view, and (D) left anterior oblique cranial view. The blue and orange arrows demonstrate the lesions within the left anterior descending artery and a diagonal branch, respectively.

**Figure 2:**
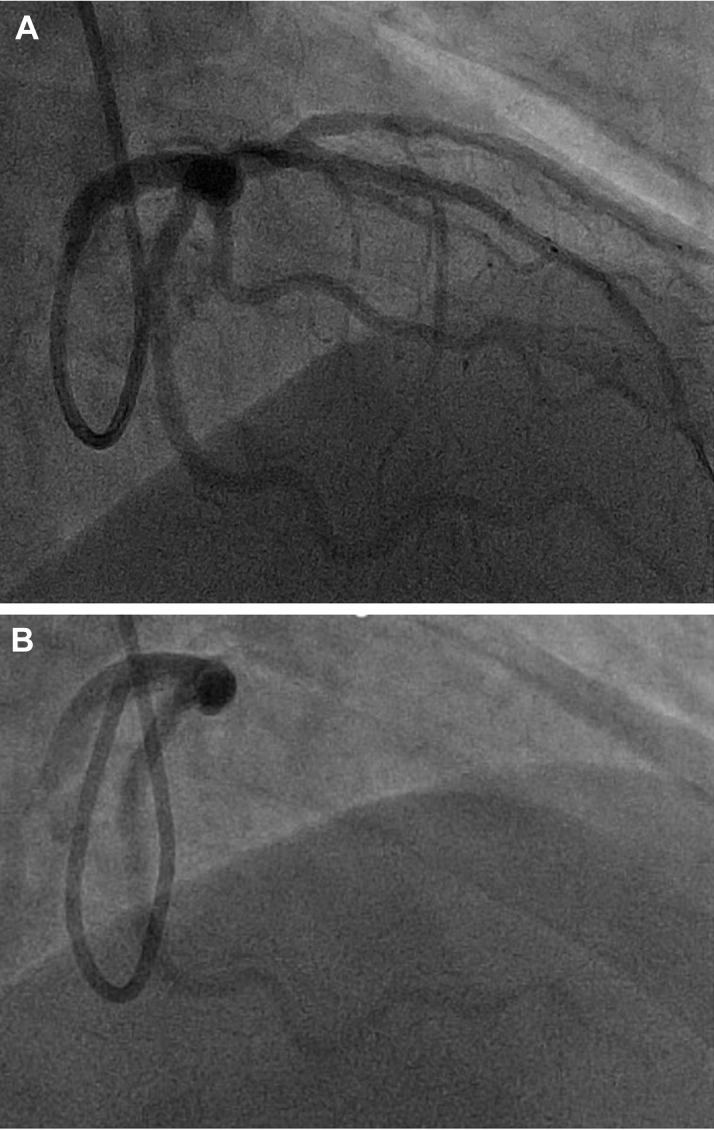
Left Coronary Angiography Before and After Wire Removal Angiography of the (A) left anterior descending artery and diagonal branch and (B) on removing the wire, thus showing the moment of aorto-ostial left main stem dissection with no antegrade flow in the left anterior descending artery.

Percutaneous coronary intervention (PCI) was performed through the right radial artery by using a 6-F sheath. A 6-F Voda left 3.5 guide catheter was used to engage the left system, and a Judkins right diagnostic catheter was used for the right coronary artery (RCA), which was free of any significant stenoses. Left coronary angiography revealed a significant lesion in the mid-left anterior descending (LAD) artery and the first diagonal (D1) branch (Figures 1A to 1D). Both the LAD artery and D1 branch were wired, and optical coherence tomography-guided PCI was performed to the D1 and LAD lesions without complication (Figure 2A).

**Figure 3:**
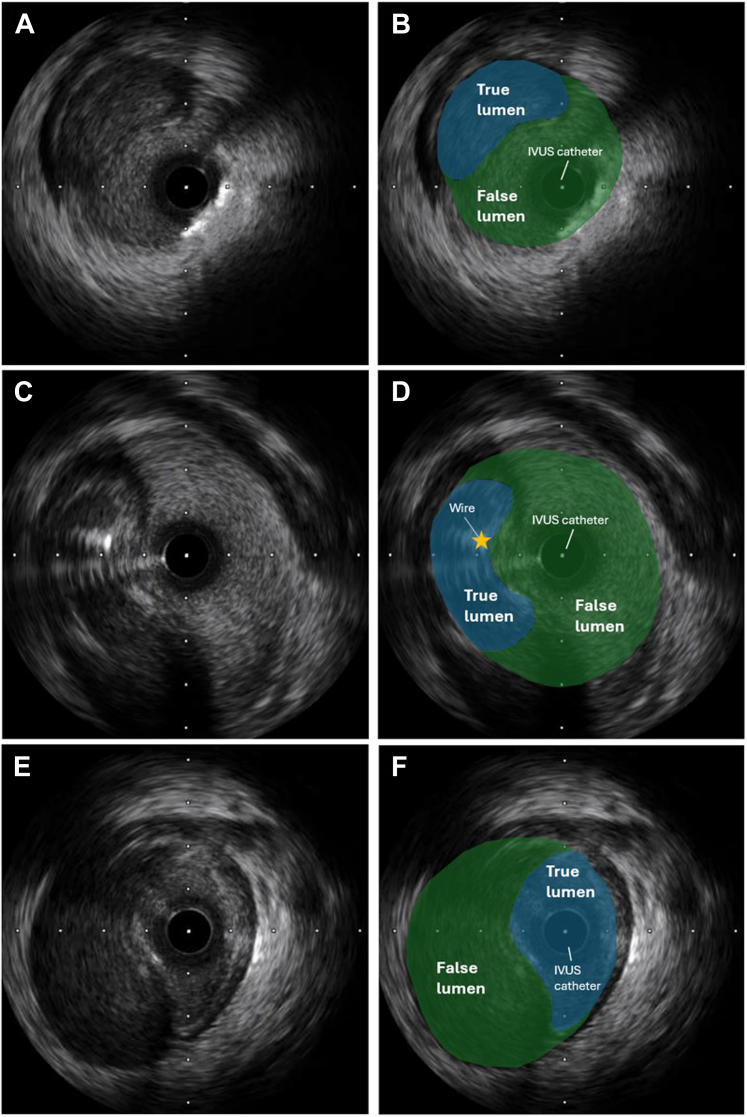
Intravascular Ultrasound Imaging demonstrating the intravascular ultrasound catheter (A and B) within the false lumen and (C and D) wiring of the true lumen. Confirmatory intravascular ultrasound was then performed (E and F).

After withdrawing the wires and setting up for final angiography, a test injection was performed to ensure guide catheter engagement. This test resulted in contrast staining within the aorta, a finding suggesting that there had been an aorto-ostial dissection (Figure 2B, Video 1). This test also demonstrated that there was no antegrade flow within the LAD artery. A Runthrough floppy (Terumo) wire was passed into the left circumflex artery, but despite numerous attempts, a further Runthrough floppy wire would not advance into the LAD artery. Antegrade wire escalation with a Gladius MG wire (Asahi Intecc) was also unsuccessful. IVUS was performed over the circumflex wire and demonstrated that the wire was in a false lumen (Figures 3A and 3B, Video 2).

**Figure 4:**
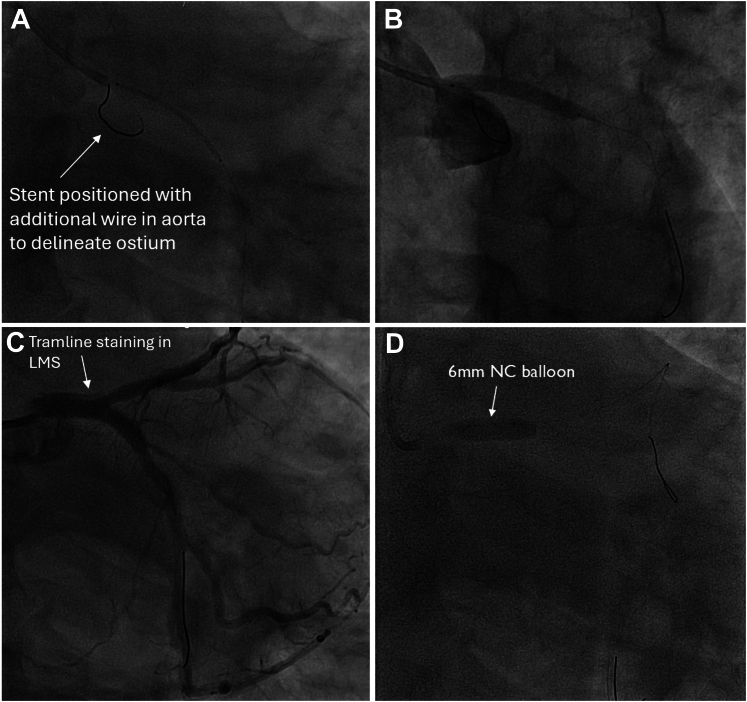
Stent Procedure (A) Demonstrating positioning of the stent with a wire in the aorta, (B) deployment of the stent, (C) return of antegrade flow within the left anterior descending artery but with residual hematoma within the left main stem (LMS), and (D) post-dilatation of the stent. NC = non-compliant.

**Figure 5:**
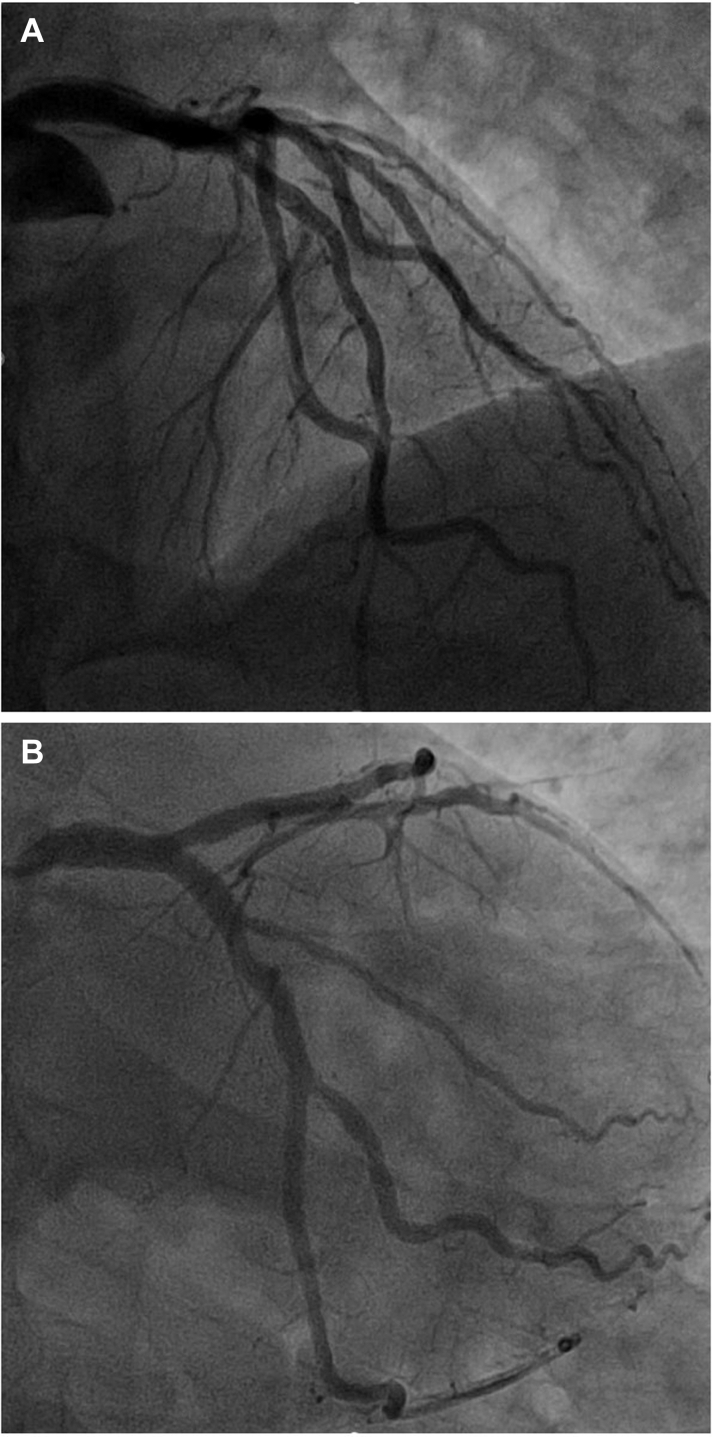
Final Angiography Imaging in the (A) posteroanterior cranial and (B) posteroanterior caudal views demonstrating antegrade flow in all coronary vessels and some residual dissection in the distal circumflex (seen more clearly in the cranial view).

The guide catheter was withdrawn slightly to disengage from the left main stem in an attempt to wire the true lumen at the origin of the dissection flap. While maintaining IVUS on the false lumen wire, a second Runthrough floppy wire was passed into the circumflex artery. On the first attempt, this wire passed into the true lumen, which was demonstrated on IVUS from the false lumen wire (Figures 3C and 3D, Video 3). To confirm the true lumen position of the new Runthrough wire, IVUS pullback was performed on the true lumen wire (Figures 3E and 3F, Video 4). The false lumen wire was now removed to simplify the procedure and avoid mistakenly stenting the false lumen. The plan was to stent the left circumflex from the proximal portion back to the ostium of the left main stem (with 1-mm aorto-ostial protrusion) to seal the dissection flap. To minimize contrast medium injections and propagation of the dissection plane, a second coronary wire was passed into the aorta to mark the position of the left main stem ostium (Figure 4A). A 4.0 × 24 mm biomatrix alpha (Biosensors International) drug-eluting stent was implanted at high pressure (Figure 4B). Angiography revealed that flow had returned within the LAD artery (Figure 4C). The portion within the left main stem was post-dilated with a 6-mm balloon (Figure 4D), with ostial flaring. The LAD artery was wired with a workhorse wire, and IVUS was attempted but could not pass easily through the stent struts of the left main stem stent. Therefore, a 3.0-mm balloon was used to dilate the stent struts at the ostium of the LAD artery. This technique allowed the IVUS catheter to pass easily. IVUS confirmed no significant residual dissection within the LAD artery (Video 5) and minor intramural hematoma within the left circumflex artery (Video 6). Final angiography showed TIMI flow grade 3 and widely patent LAD and circumflex vessels (Figures 5A and 5B, Video 7). The patient was discharged 2 days later without further complication.

## Case 2

### Background

**Figure 6:**
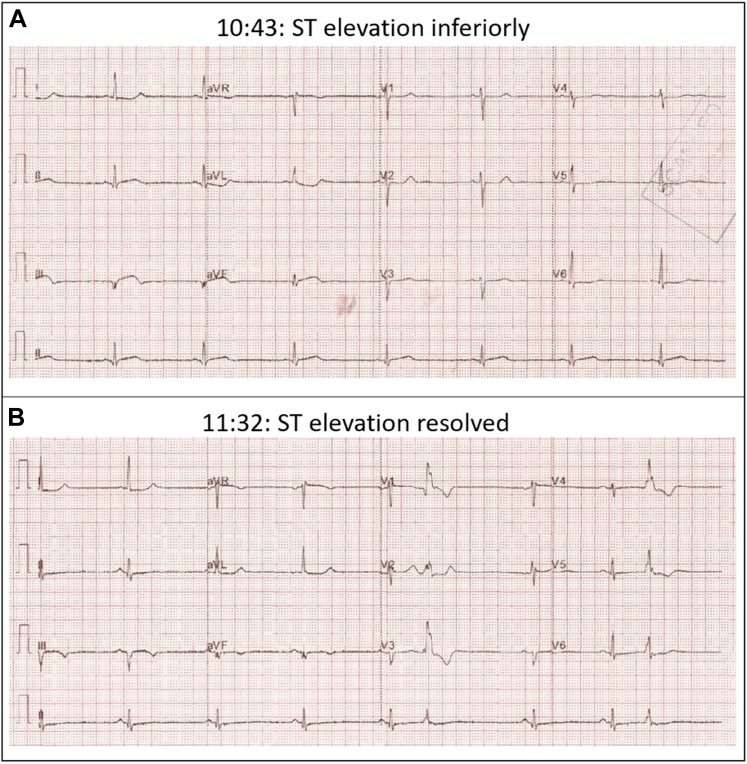
Electrocardiograms Showing Stuttering ST-Segment Elevation Electrocardiograms demonstrating transient ST-segment elevation (A) which subsequently spontaneously resolved (B).

A 44-year-old woman had a background of conservatively managed spontaneous coronary artery dissection (SCAD) in the left anterior descending and diagonal arteries in 2014 and 2016. She presented with chest pain, and serial ECGs demonstrated an aborted ST-segment elevation myocardial infarction (Figure 6). The patient’s high-sensitivity troponin level was 22,423 ng/L. In view of her ongoing chest pain and significantly elevated troponin, the patient was taken to the cardiac catheterization laboratory.

### Procedure

**Figure 7:**
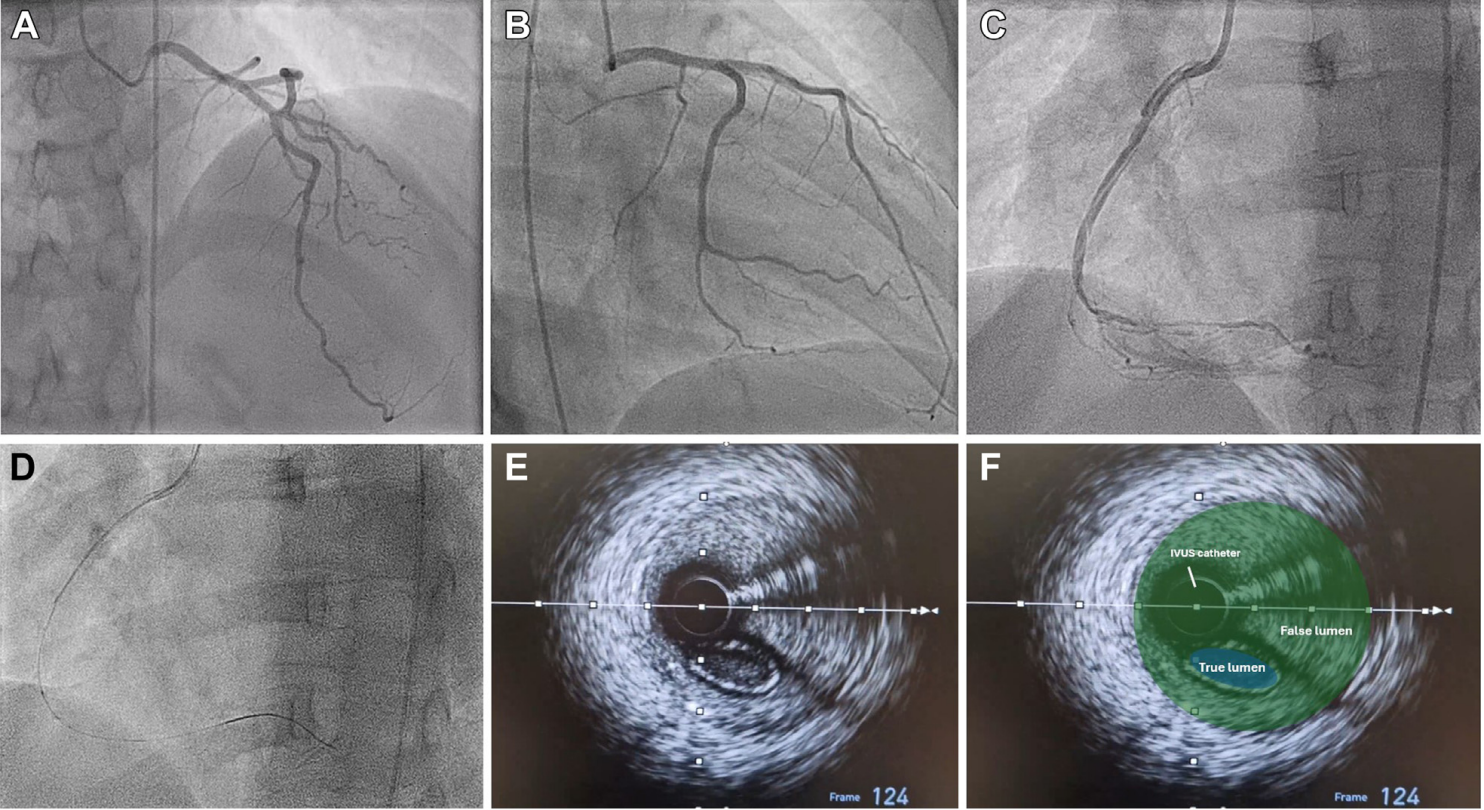
Coronary Angiography and Intravascular Ultrasound Coronary angiography demonstrating no significant disease in the (A) left anterior descending artery and (B) left circumflex artery. (C) On engagement of the right coronary artery, the catheter caused a spiral dissection. (D) Coronary wire passed into the right coronary artery with intravascular ultrasound (IVUS) performed over the coronary wire. (E and F) Intravascular ultrasound shows the wire and intravascular ultrasound catheter with a large false lumen.

**Figure 8:**
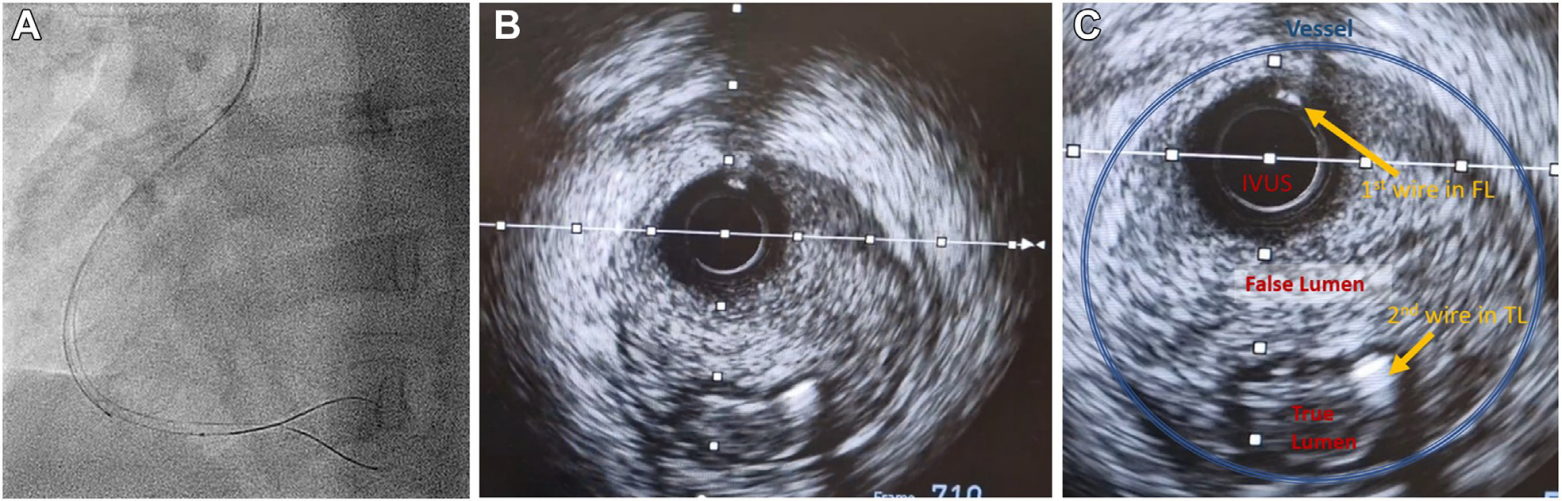
Angiography and Intravascular Ultrasound (A) Second guidewire on angiography in the true lumen (TL) on (B and C) intravascular ultrasound with the original wire in the false lumen (FL).

**Figure 9:**
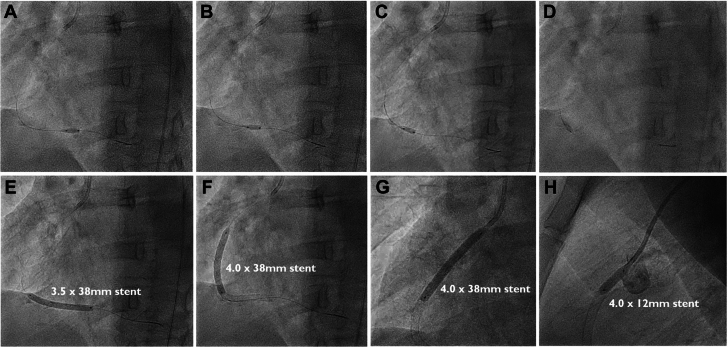
Cutting Balloon and Stent Treatment (A to D) Cutting balloon used to dissect the tunica intima to drain the intramural hematoma and facilitate stenting. (E to H) Right coronary artery stented from distal to proximal with overlapping drug-eluting stents.

**Figure 10:**
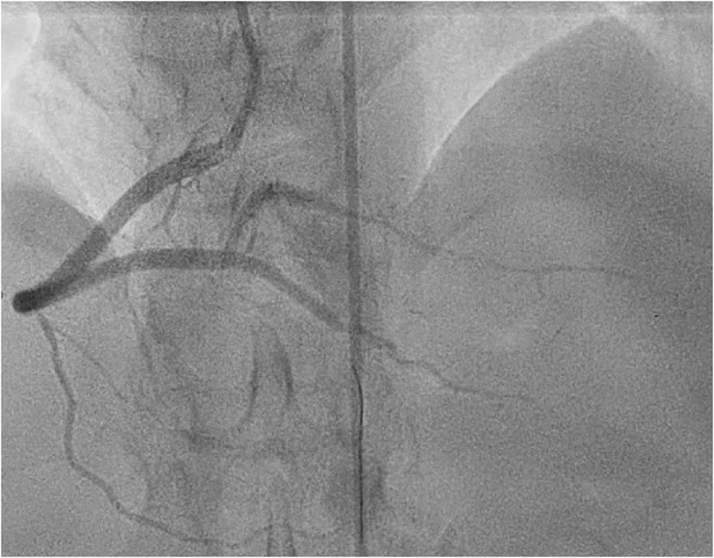
Final Angiography Imaging in the posteroanterior cranial view demonstrating a smooth, unobstructed right coronary artery with no evidence of residual dissection.

Coronary angiography was performed through the right femoral artery because of radial spasm. We used 5-F diagnostic Judkins left 3.5 (JL3.5) and right 4 (JR4) catheters (Figures 7A to 7F). The RCA was engaged using an JR4 catheter. On testing to check engagement, the catheter tip induced a spiral dissection (Figure 7C, Video 8). The vessel was wired with a Sion blue wire (Asahi Intecc), and IVUS was performed. This imaging confirmed that the wire was in the false lumen (Figure 7E, Video 9). The guide catheter was disengaged from the RCA and was maintained near the ostium while a second wire was passed into the vessel. The position of the wire (luminal or extraluminal) was then checked on live IVUS from the false lumen wire. After repeated attempts, the second wire was eventually spotted within the true lumen (Figures 8A to 8C). A 3.0-mm Wolverine cutting balloon (Boston Scientific) was then used to dissect the tunica intima intentionally to create an outlet for the intramural hematoma and thus facilitate stenting (Figures 9A to 9D). The vessel was then stented in an overlapping fashion from distal to proximal with 3.5 × 38 mm, 4.0 × 38 mm, 4.0 × 38 mm, and 4.0 × 12 mm drug-eluting stents (Figures 9E to 9H). The final stent was implanted with intentional protrusion into the aorta to seal a proximal aorto-ostial flap. Angiography revealed TIMI flow grade 3 with no angiographic evidence of residual dissection (Figure 10, Video 10), and IVUS demonstrated well apposed and expanded stents with no evidence of residual hematoma. The patient’s chest pain resolved, and she was discharged the following day. An echocardiogram revealed mild left ventricular systolic dysfunction. The patient was asymptomatic on routine review in the outpatient clinic.

## Discussion

In this case series, we demonstrate the necessary steps to perform rescue treatment of iatrogenic spiral catheter dissections. Recognition and avoidance of further contrast injections are the most important initial steps. A guidewire should then be passed into the affected vessel and IVUS performed. If the wire is in the true lumen, then the vessel can be stented. However, if the wire is in the false lumen, then a second guidewire will be required to wire the true lumen. With live IVUS imaging from the false lumen wire, the second wire is passed into the vessel and its position checked. This process is repeated until the wire is seen within the true lumen. For aorto-ostial dissections, it is important to disengage the guide catheter before attempting to wire the true lumen because the inlet of the true lumen is ostial. Once the second wire is seen within the true lumen, we recommend performing IVUS on the true lumen wire to confirm its position and to remove the false lumen wire at that stage to avoid confusion.

This procedure is not technically challenging, but the patient must remain hemodynamically stable during the process; this may require anesthetic and pharmacologic support. Two operators are ideally required, 1 passing the second wire repeatedly while the other checks the wire position on IVUS and gives verbal feedback. One limitation of the procedure is that there is an element of luck involved in wiring the true lumen, particularly in the case of large dissections. If repeated attempts fail, changing the bend on the wire may improve the likelihood of successful wiring. Another limitation is that it is reliant on the ability of the operator monitoring the IVUS to detect the relatively small echogenic guidewire within the true lumen. This may be particularly difficult to appreciate with lower-frequency 20-MHz IVUS transducers. To optimize the chances of success, we recommend using an IVUS transducer of ≥40 MHz if available. It is also important to position the IVUS transducer in a portion of the vessel where the true lumen is clearly visible, even if this is not within the proximal vessel.

Although this case series describes the treatment of iatrogenic spiral dissections, prevention is better than the cure. Maintaining catheter coaxiality, avoiding deep engagement, and checking the transduced pressure at the catheter tip before injections can avoid most dissections. The choice of catheter can also significantly affect the risk of dissection. In the left coronary system, guide catheters contribute to the majority of dissections (70%), and diagnostic catheter dissections are predominantly caused by Tiger (Terumo Medical Corp) or Amplatz catheters. For RCA angiography, diagnostic catheters account for the majority of dissections.^2^

Although many wire-out cines are taken in the cranial projection to examine for distal wire perforations, the coaxiality of the guide catheter cannot be appreciated in this view. Furthermore, removal of the wire (which may have been contributing to guide catheter coaxiality), may cause the guide to face the roof of the left main stem (as may have occurred in case 1). If there is doubt about the coaxiality of the guide, checking in the caudal view is recommended.

Coronary dissections can be categorized according to the National Heart, Lung and Blood Institute (NHLBI) classification,^3^ although a separate classification of aorto-ostial dissections has been proposed.^4^ The cases described in this report represent the highest grade (type F NHLBI classification; type III Dunning classification) because of the loss of antegrade flow and the length of dissection. The mortality from such high-grade dissections is estimated to be 6.5%.^5^

This case series shows 2 types of PCI strategy for such dissections: stenting of the inflow only (case 1) and treating the entire length of the vessel (case 2). Although the conventional wisdom is to stent from dissection inflow to normal vessel to secure the vessel in the short term, this may involve a full metal jacket that increases the risk of future in-stent restenosis. Furthermore, stenting can “milk” the intramural hematoma, thus requiring more stenting than initially planned. This issue can be somewhat mitigated by the use of a cutting balloon to dissect the tunica intima and drain the hematoma before stenting^6^ or using a subintimal microcatheter to aspirate the hematoma from within the false lumen (subintimal transcatheter withdrawal [STRAW] technique).^7^

However, given that follow-up angiography of SCAD patients has shown that 95% of cases of intramural hematoma spontaneously heal,^8^ it may be better to adopt a more conservative approach and minimize the amount of stent implanted. Thus, stenting only the inflow to secure the vessel and allowing the rest of the hematoma to heal with time may be a better approach in the long run. Further studies are required to evaluate these approaches.

**Visual Summary:**
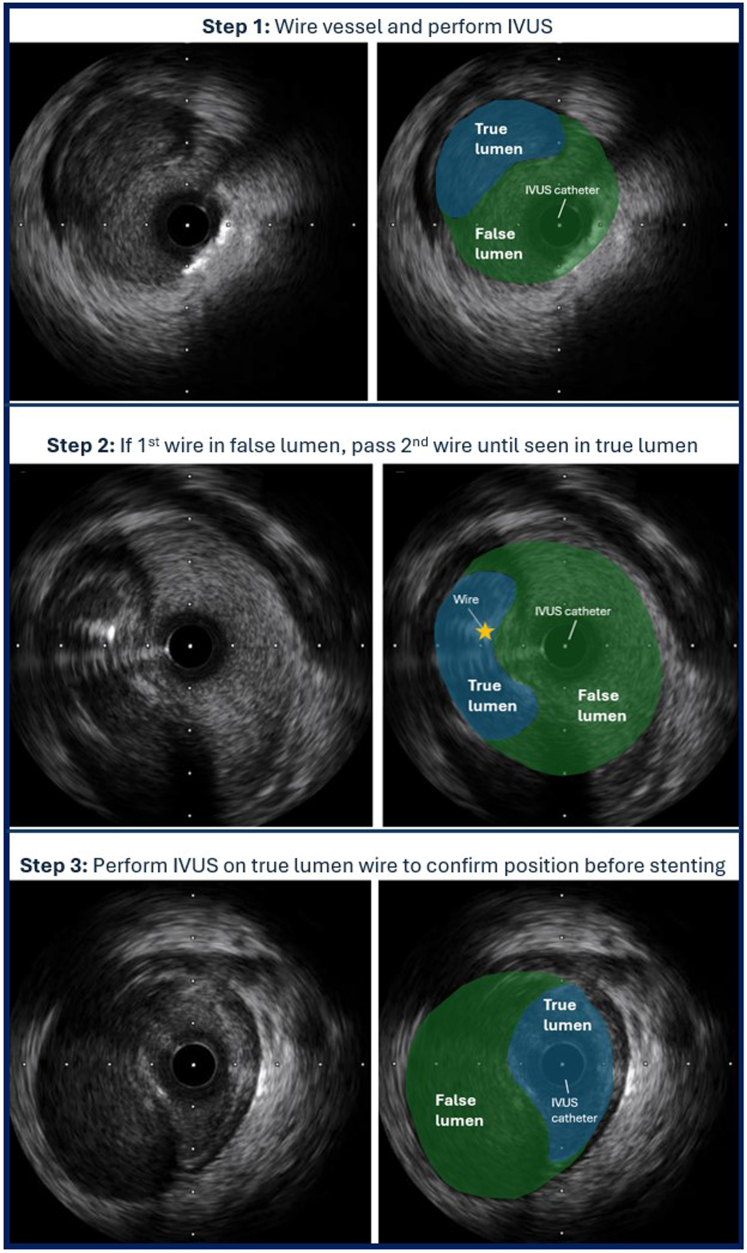
IVUS-Guided Parallel Wire Technique

## Funding Support and Author Disclosures

The authors have reported that they have no relationships relevant to the contents of this paper to disclose.

## Appendix

Video 1 Coronary Angiography Demonstrating Aorto-ostial Dissection With Staining Within The Aorta

Video 2 IVUS Performed on the Left Circumflex Wire Demonstrating That It Is in the False Lumen

Video 3 IVUS Performed From the False Lumen Wire Showing a Second Wire in the True Lumen

Video 4 IVUS Performed on the True Lumen Wire

Video 5 IVUS of the LAD and Left Main Stem After Stenting the Left Main Stem

Video 6 IVUS of the Left Circumflex and Left Main Stem After Stenting the Left Main Stem

Video 7 Coronary Angiography Demonstrating TIMI Flow Grade 3in All Vessels

Video 8 Iatrogenic Catheter Spiral Dissection Incurred on Engaging the Right Coronary Artery

Video 9 IVUS Demonstrating That the Wire Is in the False Lumen

Video 10 Final Angiography Showing TIMI flow Grade 3 With No Evidence of Residual Dissection
